# Characterizing Phage Genomes for Therapeutic Applications

**DOI:** 10.3390/v10040188

**Published:** 2018-04-10

**Authors:** Casandra W. Philipson, Logan J. Voegtly, Matthew R. Lueder, Kyle A. Long, Gregory K. Rice, Kenneth G. Frey, Biswajit Biswas, Regina Z. Cer, Theron Hamilton, Kimberly A. Bishop-Lilly

**Affiliations:** 1 Defense Threat Reduction Agency, Fort Belvoir, VA 22060, USA; casandra.w.philipson.civ@mail.mil; 2 Biological Defense Research Directorate, Naval Medical Research Center, Fort Detrick, MD 21702, USA; logan.j.voegtly.ctr@mail.mil (L.J.V.); matthew.r.lueder.ctr@mail.mil (M.R.L.); kyle.a.long8.ctr@mail.mil (K.A.L.); gregory.k.rice.ctr@mail.mil (G.K.R.); kenneth.g.frey4.civ@mail.mil (K.G.F.); biswajit.biswas.civ@mail.mil (B.B.); regina.z.cer.ctr@mail.mil (R.Z.C.); theron.c.hamilton.mil@mail.mil (T.H.); 3 Leidos, Reston, VA 20190, USA

**Keywords:** phage therapy, viral genomes, best practices, IND, high-throughput sequencing

## Abstract

Multi-drug resistance is increasing at alarming rates. The efficacy of phage therapy, treating bacterial infections with bacteriophages alone or in combination with traditional antibiotics, has been demonstrated in emergency cases in the United States and in other countries, however remains to be approved for wide-spread use in the US. One limiting factor is a lack of guidelines for assessing the genomic safety of phage candidates. We present the phage characterization workflow used by our team to generate data for submitting phages to the Federal Drug Administration (FDA) for authorized use. Essential analysis checkpoints and warnings are detailed for obtaining high-quality genomes, excluding undesirable candidates, rigorously assessing a phage genome for safety and evaluating sequencing contamination. This workflow has been developed in accordance with community standards for high-throughput sequencing of viral genomes as well as principles for ideal phages used for therapy. The feasibility and utility of the pipeline is demonstrated on two new phage genomes that meet all safety criteria. We propose these guidelines as a minimum standard for phages being submitted to the FDA for review as investigational new drug candidates.

## 1. Introduction

Phage therapy, the use of bacteriophages to treat bacterial infections, especially in combination with traditional antibiotics, is recognized as a promising strategy to combat multi-drug resistant (MDR) infections [1]. Although phages are generally considered safe [2,3,4], guidelines for genetic safety assessments of phages prior to clinical use are non-existent. Currently, there is no general approval process for phage therapy in the United States. The Food and Drug Administration (FDA) can grant emergency investigational new drug (eIND) status for phage cocktails in compassionate care cases, however this process requires a request from a medical doctor and protocols remain case-by-case. The lack of guidelines presents one limiting factor for advancing phages as therapeutic agents along the regulatory pipeline. To address this, we present a characterization workflow that implements best-in-field tools to systematically evaluate genetic safety of phage candidates for therapeutic applications. The protocol presented is the minimum standard used by our team to generate IND-enabling data and submit phage therapeutics for FDA approval.

Phage biology is an enormous field with topics ranging from viromes in the sea [5] and human gut [6], to genetic engineering [7], to therapeutic utility and countermeasure development [3]. The number and diversity of discovered bacteriophages is increasing at a rapid rate, especially with respect to viral discovery efforts using high-throughput sequencing. The number of complete phage genomes deposited in the NCBI Genome database has more than doubled in the last three years. As a result of diversity in investigative studies, rigor for sequencing, assembling, finishing and manually polishing phage genomes is reported at varying levels in literature depending on intended use [8,9,10,11,12]. At the assembly stage, algorithmic success often depends on empirically derived heuristics which help overcome complicated repeat patterns in real genomes, random and systematic error in sequencing reads and limitations in computational power. In order to mitigate potential bias introduced by a single assembly algorithm, it is typically necessary to employ a consensus approach utilizing multiple assemblers. This is also true for gene-calling and annotation, which can be performed using fully-automated single-platform tools such as Rapid Annotation using Subsystem Technology RAST [13]. While these platforms democratize genomics and offer efficiencies for first-glance solutions, relying on these tools alone introduces high risk for inaccurate safety assessment. For instance, inaccuracies can arise due to potential misinterpretation of unreliable data that propagate throughout public databases. Despite existing methods to identify start sites and directionality, resolve ends, and predict lifestyle for phages, the criteria employed in these methods are loosely controlled. In this study, we delineate key analytical checkpoints where manual intervention is necessary for achieving standards set forth for genomes used in therapeutic applications. The checkpoints fall within two categories: obtaining a high-quality genome and robust assessment of genetic composition.

Considering genome quality there are standards defined for viral sequences with respect to the level of completeness for desired downstream applications [14]. The recommended category for viruses used in animal models for vaccine development, and by extension phage therapy, is “Finished”. Finished status is defined as a single consensus sequence representing 100% of the genome with all open reading frames (ORFs) identified and population diversity, or lack of population diversity as an indicator of purity, of the sequence verified via deep coverage. Phage isolation, sample preparation and rationale for sequencing technology will not be discussed in detail here. However, it is important to note that any contaminants introduced by laboratory protocols, such as bacterial host remnants from phage expansion and those inherent to nucleic acid sample preparation and high-throughput sequencers, can negatively impact the ease and accuracy of obtaining a high-quality genome. As such, contaminant identification is presented as a fundamental step in the safety assessment framework.

In addition to genome quality, properties of “safe” phage therapy candidates have been discussed [15]. Primary determinants for therapeutic selection include: antibacterial virulence, lifestyle, and the absence of deleterious genes. A phage’s host-range and antibacterial virulence (efficacy) are evaluated using experimental techniques during initial selection; however, the presence or absence of deleterious genes is analyzed by computational methods. Likewise, phage lifestyle is analyzed first by experimental methods in the laboratory, then additionally evaluated by computational methods. Phage Classification Tool Set (PHACTS) [16] is an example of a tool that can be used for computational analysis of lifestyle. Lifestyle of a phage can be classified by two different states, lytic or lysogenic, with the former being the necessary state for phage therapy candidates. In a lysogenic state, a phage integrates its DNA into the DNA of the host to become a prophage, rendering itself dormant and suppressing the typical anti-bacterial properties exhibited during a lytic state. Additionally, a prophage could provide bacteria with a mutualistic relationship, increasing the fitness of the host and thereby decreasing the effectiveness of phage therapy. Mechanisms underlying lysogeny are largely mediated by the presence of a functional phage-encoded enzyme, integrase. Although temperate phages have been found viable to deliver some non-lytic antimicrobial treatments [17,18,19], the risks associated with lysogenic phages or prophages are reason enough to avoid using them in phage therapies. Hence, the presence of an integrase gene is undesirable and nullifies a phage’s candidacy for therapy unless methods are developed to determine if the integrase is indeed non-functional. Similarly, as some specific bacterial infections can exhibit enhanced virulence mediated by phage-encoded toxins (e.g., Shiga toxin, diphtheria toxin and cholera toxin), toxins must be screened for as well. Detecting any of the following by genetic screening would immediately disqualify a phage for therapeutic use: genes that encode virulence factors, antimicrobial resistance, toxins, or transducable elements.

**Figure 1:**
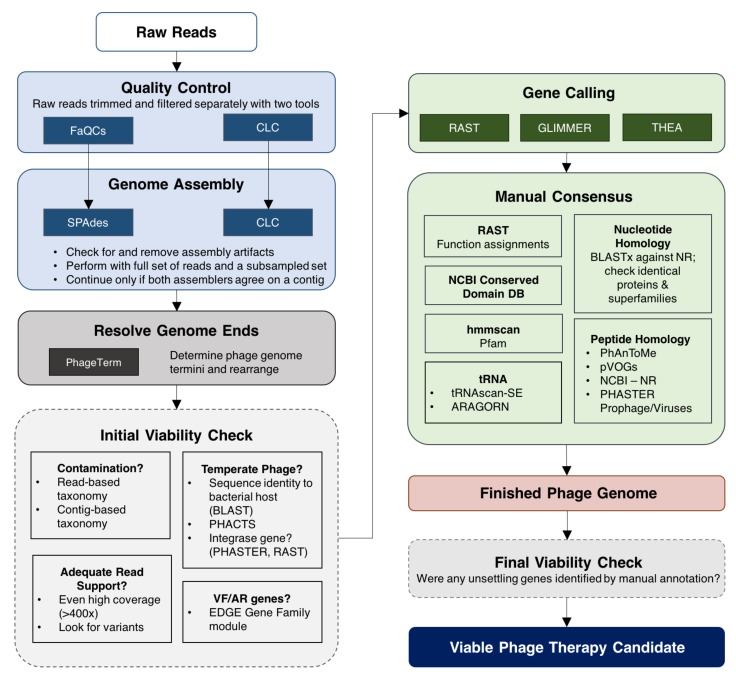
Phage characterization workflow. This pipeline is a simplified representation of tools and methods used to obtain high-quality phage genomes that are deemed viable phage therapy candidates. The pipeline begins with raw reads sequenced on an Illumina machine. To reduce potential bias introduced by bioinformatics tools, quality control and genome assembly are performed using two pipelines in parallel. The final genome sequence is obtained after resolving genome ends. Key viability checkpoints are outlined with dashed borders. In the initial viability check, phages are assessed for problematic genes (antimicrobial resistance (AMR), virulence factors (VF), toxins) and lifestyle. If a candidate passes the initial viability check, a combinatorial approach is applied to identify open reading frames followed by rigorous manual annotation. A final check is performed after completing annotation. Phage candidates that pass the final check point are considered safe for potential use in humans.

To date, phage therapy approved under eIND bypasses conventional in vivo safety studies. This necessitates a rigorous safety screening platform to safeguard patients. It remains unclear whether commercialization of phage therapy will facilitate wide-spread use, personalized treatment, or rely on broad-host-range cocktails. For any case, we present a characterization pipeline as a guideline for minimum assessment standards of phages administered to patients (Figure 1). The pipeline, which has been developed and used by our team to prepare phage genomic data in support of IND submission, includes methods to: (i) obtain a high quality whole genome sequence; (ii) identify open reading frames (ORFs); (iii) annotate genes with a consensus function identified across tools; (iv) search for deleterious genetic markers; (v) verify that the sequence is representative of the population; and (vi) perform contaminant analysis. We demonstrate the pipeline’s utility by delivering two finished phage genomes that meet safety criteria for phage therapy.

## 2. Materials and Methods

### 2.1. Phage Isolation and Genomic DNA Extraction

The phages sequenced in this study were isolated from environmental samples by routine isolation techniques [20]. Then they were triple plaque-purified on their respective hosts and inoculated at a multiplicity of infection (MOI) of 0.1 into 100 mL cultures of their respective host bacteria for amplification at 37 °C in preparation for sequencing. Upon lysis of the bacterial cells, the lysate for each phage was filtered through a 0.22 µm filter, DNAse-treated in presence of MgCl_2_ to degrade DNA that is not protected by viral capsid (e.g., host DNA), Proteinase K- and sodium dodecyl sulfate-treated to inactivate DNAse and disrupt capsid, followed by Phenol-chloroform-isoamyl alcohol extraction, debris removal and polyethylene glycol precipitation of naked DNA in the presence of salt (NaCl) [21]. The nucleic acid pellet was washed with 80% alcohol and dissolved in deionized distilled water before RNAse treatment. The RNAse-treated samples were extracted one more time with Phenol-chloroform isoamyl alcohol and DNA precipitated in presence of absolute alcohol. Finally, the DNA pellet was washed with 70% alcohol before being suspended in deionized distilled water. The resulting phage genomic DNA was subjected to rigorous internal quality control testing, including agarose gel electrophoresis to ensure high molecular weight (indicative of relatively non-sheared, intact genomic DNA), restriction enzyme digests to assess potential genome modifications that prevent manipulability by sequencing library protocols, Qubit measurements (Thermo Fisher Scientific; Waltham, MA, USA) for concentration and Nanodrop measurements (Thermo Fisher Scientific) for purity (optical density 260/230 ratio).

The above protocol was used strictly for preparation of phages for sequencing efforts whereas for clinical use our phage preparations have been conducted via CsCl gradient ultracentrifugation, as in Schooley et al [3], to completely remove any host material such as naked bacterial DNA.

### 2.2. Library Preparation and Sequencing

Sequencing libraries were constructed using the Accel-NGS^®^ 2S Plus library kit (Swift Biosciences, Ann Arbor, MI, USA) with a slight modification. Briefly, 250 ng of genomic DNA was fragmented using the Covaris M220 (Covaris, Inc, Woburn, MA, USA). Instrument parameters were the factory settings for Illumina TruSeq (350 bp). The sheared gDNA was subjected to a double-sided size selection using AMPure XP beads (Beckman-Coulter, Brea, CA, USA). Selection ratios were 0.75×/0.6×. Size-selected DNA was then used as input for 2S Plus. Library fragments were not amplified using PCR. All libraries were quality checked using the Agilent BioAnalyzer (Agilent Technologies, Santa Clara, CA, USA) and quantitated using the NEBNext^®^ Library Quant kit (New England Biolabs, Inc., Ipswitch, MA, USA). Prior to sequencing, individual libraries were diluted to 2 nM concentration and pooled. Sequencing was performed on a MiSeq (Illumina, Inc., San Diego, CA, USA) using 2 × 300 v3 chemistry. Raw sequencing reads were deposited in NCBI’s Sequence Read Archive (SRA) under SRA accession numbers (SRR6764339, SRR6764268).

### 2.3. Genome Assembly

For each sample, two pipelines were run in parallel for quality control (QC) and assembly (Figure 1). Results from both pipelines were compared to identify a single consensus sequence with high confidence. Specific parameters employed are listed in Supplemental Table S1. If there was not 100% nucleotide identity between the results of the largest contig in both assemblers, the reads were mapped to both contigs in order to manually verify the fidelity of the assembly. Both pipelines implemented the following tasks in order: (1) raw data were processed for QC; (2) all reads that pass QC in Step 1 were assembled de novo; (3) 50,000 to 100,000 quality-controlled reads from Step 1 were subsampled then assembled de novo. The first pipeline combined publicly available tools: FaQCs for QC [22], seqtk for subsampling [23] and SPAdes (version 3.5.0) for assembly [24]. The second pipeline includes NGS Core Tools from CLC Genomics Workbench (version 10, Qiagen, Redwood City, CA, USA). Both FaQCs and CLC’s quality trimmer were set to trim reads to Q30 and remove reads less than 50 bp in length. CLC’s quality trimmer was set to remove any reads containing more than two ambiguous bases while FaQCs was set to remove reads with more than two consecutive ambiguous bases. In addition, the way quality trimming is implemented differs between the two pipelines. FaQCs utilizes BWA-style trimming [14] while CLC’s quality trimmer utilizes a modified-Mott algorithm. These differences make CLC’s quality trimming more stringent. Between the two pipelines, four de novo assemblies were performed for each sample: SPAdes-all reads, SPAdes-subsampled, CLC-all reads, and CLC-subsampled. Subsampling was performed to achieve 80–100× coverage of the genome. If a genome size was unknown, 50,000 reads were subsampled; a maximum of 100,000 reads is recommended for the initial assembly of unknown phage genomes [8]. When sampling paired-end reads, half of the reads should be obtained from each file. SPAdes assembly was performed using default settings whereas CLC assembly parameters were default except for word size, which was set to 64. Assembly artifacts were identified and removed (i.e., 127 bp artificial overlaps at the ends of SPAdes assembled contigs). If the largest contig generated by two different assemblers presented 100% nucleotide identity, regardless of start site or orientation, the resulting sequence proceeded to downstream analysis. Otherwise, all reads were mapped back to the contig to determine genome ends versus sequencing artifacts manually by visualizing read support using CLC. The resultant assembly size is also compared to the range of genome sizes for that particular virus family to make sure that it is relatively consistent with the expected value (virus family being determined either by morphological characteristics, closest sequenced relatives, or both).

### 2.4. Resolving Genome Ends

The genomic termini and phage packaging strategy were determined using PhageTerm [25]. Briefly, FaQCs processed reads were aligned to the putative phage genome and read build-ups, indicative of over-represented fragment ends, were identified. PhageTerm uses the starting position coverage (SPC) and the coverage in each orientation (COV) to calculate $\tau =\frac{SPC}{COV}$ in each direction for each nucleotide. This metric is used to determine the location of genomic termini as well as classify it as one of the following: 5′ *cos*, 3′ *cos*, direct terminal repeat (DTR) (short), DTR (long), headful (with or without *pac* site detected), Mu-like, or unknown. It automatically rearranges the genome sequence accordingly. All genomes were checked for non-terminal nucleotides with significant *p*-values and aberrant coverage patterns. When a phage genome’s termini could not be determined, or in the case where a phage genome had no consistent biological termini (circularly permuted phages), the start site was selected based on the presence and orientation of terminase genes as previously described [26] in order to allow easy comparative analysis of similar phages. If no large terminase gene was found, the start site was selected based on alignment with the closest reference in GenBank.

### 2.5. High Quality Genome Checkpoint

After ends were resolved and orientation was set, the final genome sequence was validated for quality. To validate the genomic sequence, quality-controlled reads were mapped back to the sequence to ensure it was well-supported by sequencing data. Assembly validation was performed in CLC using clc_mapper with default settings but can be performed at the command line interface using BWA. Three metrics were considered: percentage of reads mapping to the genome, average whole genome coverage and lowest coverage. Assemblies were considered validated if >90% of reads mapped back to the phage genome. Additionally, in order for genomes to proceed from this checkpoint, average whole genome coverage and lowest coverage were at least 100× for complete genomes and ~400× for finished genomes [14]. “Complete” and “finished” genome definitions used here are as defined by Ladner et al.; specifically, complete viral genomes have the whole sequence fully resolved, including ends, whereas finished viral genomes have a complete sequence plus a minimum of 400–1000× coverage depth to resolve population-level variations [14]. Genomes with <400× coverage can proceed through the pipeline, however appropriate detection of single nucleotide polymorphisms (SNPs) in the population requires the deeper coverage metric. Additional sequencing can be performed to obtain more reads for population-level validation of complete phage genomes.

### 2.6. Phage Lifestyle Checkpoint

Temperate phages do not proceed as viable therapeutic candidates in our pipeline. Therefore, an important checkpoint involves identifying phages that have the potential for temperate behavior. Complete genomes were submitted to RAST [13] and PHAge Search Tool Enhanced Release (PHASTER) [27] for baseline gene calling and functional annotation. Output from these tools was parsed for the presence of “integrase.” Additionally, PHACTS was utilized to determine if a phage’s overall proteome resembled that of a temperate phage. Briefly, PHACTS is a tool that utilizes a Random Forest classifier to predict phage lifestyle, bacterial host and phage family by comparing proteins from the query to those of phages within the PHACTS database [16]. PHACTS analysis yields a statistically-based score that predicts the likelihood that a phage is prone to a temperate versus lytic lifestyle. Any indicators of temperate behavior result in rejection of the phage candidate.

### 2.7. Specialty Genes Checkpoint

An initial viability check is performed to identify the presence of toxins, virulence factors, or antimicrobial resistance genes. This step is performed on reads and contigs using the EDGE Bioinformatics Gene Family module (Appendix A). Read-based functional profiling was performed on FaQCs processed reads. ShortBRED (v0.9.4M) searches reads for similarity to antibiotic resistance genes found in three databases: Antibiotic Resistance Genes Database (ARDB) [28], Resfams antibiotic resistance functions [29] and Virulence Factors of Pathogenic Bacteria (VFDB), downloaded December 2015. [30]. Contigs from the SPAdes-all reads assembly were also searched for problematic genes. ORFs were predicted in all contigs >700 base pairs in length using Prodigal [31]. ShortBRED was used to search predicted ORFs against VFDB. Additionally, the Resistance Gene Identifier (RGI), v3.1.1 with database from July 2016, was used to search predicted ORFs against the Comprehensive Antibiotic Resistance Database (CARD) [32]. Databases and sources are listed in Table 1. Default parameters were used for all tools. Detecting any positive hits in this checkpoint renders a phage unfit for therapeutic use. It is important to note that Prodigal is a gene calling algorithm designed to predict ORFs in prokaryotes, thus it is expected to perform well for those organisms. Prodigal is used in this step because it is the gene caller embedded in the EDGE Bioinformatics Gene Family module. We have found this method appropriate for fast candidate viability checks, however none of the gene calls from Prodigal are used for annotation since GLIMMER3 outperforms Prodigal for predicting ORFs in phages. Users are not confined to relying on Prodigal as the method for identifying ORFs or for specialty gene analysis.

### 2.8. Contaminant Analysis

Reads and contigs were analyzed for host and laboratory contamination using EDGE Bioinformatics software [33]. Taxonomy Classification was performed on FaQCs processed reads using four tools: GOTTCHA (version 1.0b) [34], Kraken (version 0.10.4-beta) [35], MetaPhlAn (version 1.7.7) [36] and BWA-mem (version 0.7.9) [37] mapping to RefSeq. Contigs from SPAdes-all reads assembly were classified by aligning contigs to NCBI’s RefSeq database using BWA-mem. All programs were run using default parameter settings in EDGE Bioinformatics (Appendix A) [38]. Any contigs >700 bp with >5× coverage obtained in subsampled assemblies were also analyzed by megablast against nr/nt databases. Samples were considered free from contamination if the total assembly size was close to the range of genome sizes within that particular virus family and if less than 5% of reads mapped to host reference genomes.

### 2.9. Gene Calling and Functional Annotation

Baseline gene predictions and functional annotation were obtained from Classic RAST (Virus domain; genetic code 11; FIGfam version Release70) [13]. Putative ORFs were also predicted using the command line version of GLIMMER-3 (v3.02) [39] and the phage-specific gene caller THEA [40]. Start- and stop-site coordinates were compared for the two approaches and disagreements were considered during manual gene assignments. Annotation was performed manually. The nucleotide sequence for each predicted ORF was queried by BLASTx against NCBI’s non-redundant (nr) protein sequences. Peptide sequences for each predicted ORF also underwent homology searches using BLASTp against nr, PhAnTOME [41], pVOGs [42] and the PHASTER Prophage/Virus databases [27]. The following threshold values were applied in general. Putative ORFs with 50–70% sequence identity [43] to a given gene were assigned “putative.” When peptide sequences exhibited low identity (less than 50% [44]), protein sequences were also submitted for the analysis of hidden Markov models by hmmscan [45] against the Pfam database [46] and NCBI’s Conserved Domain Database. Consensus gene functions were assigned to ORFs manually. tRNAs were identified using tRNAscan-SE [47] and ARAGORN [48]. Specifically, for putative ORFs with identity to potentially harmful gene products, we set a lower threshold (30%) so as to increase sensitivity and err on the side of safety. This threshold was chosen based on the work of Joshi and Xu, in which it is stated that at this threshold the chance for a pair of proteins to share any of the three GO categories at high levels would be 50% or less [43].

## 3. Results

We present two finished phage genomes that pass all safety checkpoints in the phage characterization workflow and which we have subsequently deposited in GenBank. The two phages, Pseudomonas phage vB_PaeP_130_113 (GenBank accession MH107770) and Staphylococcus phage vB_SauM_0414_108 (GenBank accession MH107769), were selected based on the clinical relevance of their bacterial hosts, *Pseudomonas aeruginosa* and *Staphylococcus aureus*, respectively and demonstrated antibacterial efficacy against clinical isolates in house. *Pseudomonas aeruginosa* and *Staphylococcus aureus* are two of the twelve antibiotic-resistant priority pathogens according to the World Health Organization (WHO); carbapenem-resistant *Pseudomonas aeruginosa* falls within the Priority 1 CRITICAL category. Both pathogens are also categorized as ESKAPE pathogens (*Enterococcus faecium*, *Staphylococcus aureus*, *Klebsiella pneumoniae*, *Acinetobacter baumannii*, *Pseudomonas aeruginosa*, and *Enterobacter species*), microbes responsible for the majority of hospital-acquired antimicrobial-resistant (AMR) infections, by the Infectious Diseases Society of America [49,50]. Results are presented according to key checkpoints in the phage characterization workflow.

### 3.1. Sequencing and Assembly Statistics

The workflow begins with two pipelines that perform Quality Control and Genome Assembly in parallel, as depicted in Figure 1. There are several QC and assembly checkpoints during this process:

1. Check reads for quality, length, nucleotide composition, ambiguous nucleotides.
2. Do assemblies agree? If no, is there adequate read-support to resolve differences?
3. Is the largest contig a phage sequence? If no, consider contamination analysis.
4. Identify (and later, characterize) all contigs >700 bp with >5× coverage.

Both samples retained 94–98% of reads following quality control (Table 2). For each sample, de novo assemblies were performed by SPAdes and CLC using all quality-controlled reads as well as a subsampled set of quality-controlled reads (four assemblies per sample; see Methods). For the Pseudomonas phage vB_PaeP_130_113 sample, all four de novo assemblies yielded a single contig that was identical among them and therefore proceeded to downstream analysis. The closest relative to Pseudomonas phage vB_PaeP_130_113 is Pseudomonas Phage DL62 (94% query coverage, 94% identity). In contrast, assemblies of randomly subsampled reads, hereafter referred to as “subassemblies”, were required to obtain contigs with 100% sequence identity for the Staphylococcus phage vB_SauM_0414_108 sample. These differences highlight the utility of a multipronged approach for obtaining a consensus sequence with high confidence. Reads were mapped back to Staphylococcus phage vB_SauM_0414_108 contigs for assembly validation and the differences observed in assemblies using all reads were deemed artificial overlaps introduced by the algorithms. The validated consensus sequence used for Staphylococcus phage vB_SauM_0414_108 in next steps was the largest contig that presented 100% nucleotide identity from the two subassemblies. The closest relative to Staphylococcus phage vB_SauM_0414_108 is Staphylococcus phage K (95% query coverage, 99% identity). SPAdes all reads assembly contained a 751 bp contig. This contig was disregarded due to low coverage (<1.5×) and because it had no significant sequence similarity when queried (megablast) against the NCBI nr database.

### 3.2. High Quality Genomes

The next step in the workflow is the determination of termini position and packaging strategy and involves the following checkpoints:

5. Are genome ends resolved?
6. Is genome supported by adequate even coverage?

Reads that passed FaQCs and the consensus sequence obtained from genome assembly were submitted for analysis using PhageTerm software. Both phages contain direct terminal repeats (DTRs). Pseudomonas phage vB_PaeP_130_113 contains a short 463 bp DTR and Staphylococcus phage vB_SauM_0414_108 has a long DTR spanning 10,296 bp (Table 3). Coverage over the DTR region was approximately twice that of the rest of the genome for both phages. The metric τ, indicative of sudden coverage peaks, also supports the presence of DTRs in both genomes. Finally, read mapping was performed to validate the rearranged genomes using all CLC quality-controlled reads and CLC aligner. The final size of Pseudomonas phage vB_PaeP_130_113 is 44,205 bp with 846.5× average whole genome coverage. The final size of Staphylococcus phage vB_SauM_0414_108 is 151,627 bp with 508.6× average whole genome coverage.

### 3.3. Phage Lifestyle

The primary objective of the phage lifestyle step is to identify temperate phages since they are not pursued past this point in our pipeline and involves the following checkpoints:

7. Do any predicted ORFs present sequence identity to known integrase(s)?
8. Do classification algorithms (i.e., PHACTS) bolster confidence?

Genomes were submitted to RAST and PHASTER for rapid preliminary gene calling and annotation. The RAST-generated Genbank file and the PHASTER-generated details.txt file were parsed (using grep) to identify the presence of integrase. In addition, the phage proteomes were analyzed using PHACTS, a computational classification algorithm trained to predict phage lifestyles. Integrase genes were not identified in either genome. In line with this, PHACTS predicted <40% probability that these two phages would exhibit temperate behavior (Table 4). We applied PHACTS analysis to our genomes along with the closest relative for each sample and two phages with integrase. Lytic scores were >0.59 for Pseudomonas phage vB_PaeP_130_113 and its closest relative (Pseudomonas phage DL62, GI:KR054031) as well as Staphylococcus phage vB_SauM_0414_108 and its closest relative (Staphylococcus phage K, GI:KF766114.1). To contrast this, we also present phages with integrases in their genomes (Pseudomonas phage vB_PaeS_PMG1, GI:NC_016765; Staphylococcus phage phiSaus-IPLA88, GI:NC_011614.1) and respective lytic scores of 0.42 or less. These results demonstrate reliable PHACTS predictions for phages with known lifestyles. Taken together, these results strongly suggest that Pseudomonas phage vB_PaeP_130_113 and Staphylococcus phage vB_SauM_0414_108 are likely lytic phages.

### 3.4. Specialty Genes Checkpoint

The next checkpoint involves genes with potential deleterious effects:

9. Are any toxins, virulence factors, or antimicrobial resistance genes detected?

To answer this question, all reads and all annotated coding sequences from contigs were profiled for antimicrobial resistance genes and virulence factors. This analysis was performed using the EDGE Bioinformatics Gene Family module, however the tools and databases are open source and can be run by command line (Table 1). The ShortBRED algorithm was used to perform a targeted search for unique and specific signatures found in four specialty gene databases (Table 1). For read-based analysis ShortBRED searches the Antimicrobial Resistance Database (ARDB), Resfams and the Virulence Factor Database (VFDB). For contig-based analysis, Prodigal performs gene calling, ShortBRED searches coding sequences against VFDB and the Resistance Gene Identifier (RGI) searches the Comprehensive Antibiotic Resistance Database (CARD). By these methods, Pseudomonas phage vB_PaeP_130_113 and Staphylococcus phage vB_SauM_0414_108 were found to encode zero hits to any known problematic specialty gene targets at the read and contig levels.

### 3.5. Contaminant Analysis

Another key element in the viability check is detection of potential contaminants, or transducing activity which can present similarly as contamination, and this analysis involves the following three checkpoints:

10. What percentage of reads are classified as phage?
11. What is the total assembly size?
12. What percentage of reads map to non-phage contigs from the assembly?

Two approaches were applied to assess contamination or potential transducing ability in a quantitative manner. First, all reads and all assembled contigs were analyzed using the Taxonomy Classification module in EDGE Bioinformatics. Parameters for quality trimming in our local instance of EDGE Bioinformatics software were updated to reflect the FaQCs and SPAdes parameters described in the Methods and Appendix A. The second approach is classification-independent: calculate the percentage of reads that map to non-phage contigs and considered total assembly size. This second measurement is calculated as follows: (1) determine the phage contig(s) versus non-phage contigs through taxonomic classification as described in Section 2.8, *Contaminant Analysis*; (2) calculate the number of reads mapped to the phage contig; (3) subtract the number obtained in Step 2 from the number of reads mapped to whole genome assembly; and (4) calculate the percentage of host sequence (i.e., the number of reads mapped to non-phage contigs divided by the total number of quality-controlled reads multiplied by 100).

**Figure 2:**
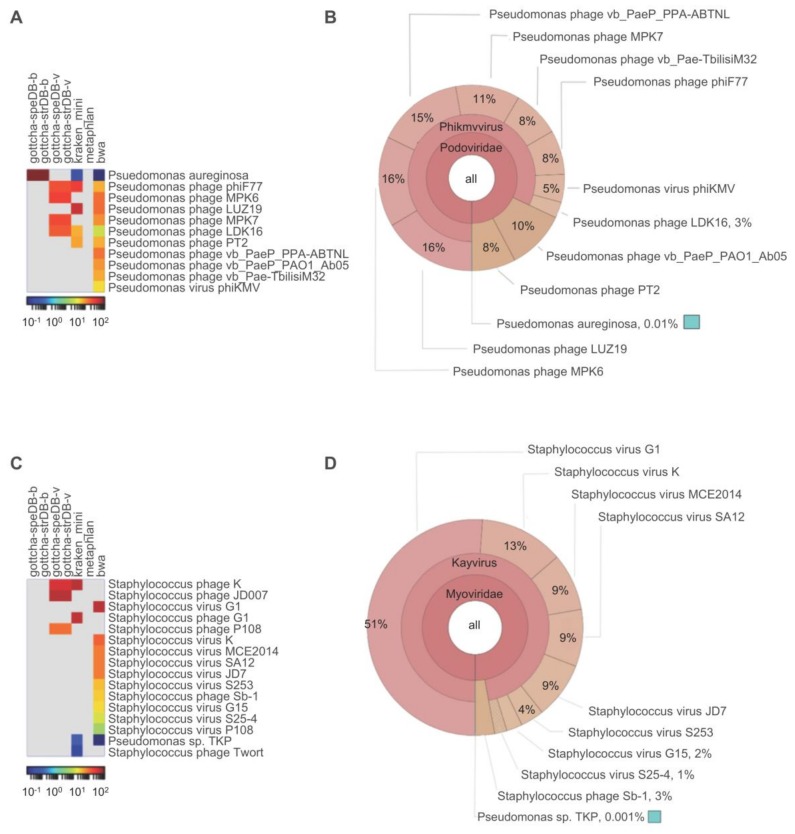
Contaminant analysis using read-based taxonomy classification. Read-based taxonomy results are presented for Pseudomonas phage vB_PaeP_130_113 (**A**,**B**); and Staphylococcus phage vB_SauM_0414_108 (**C**,**D**). Taxonomy results for all classification tools (relative abundance) using all reads that pass QC are presented as heatmaps (**A**,**C**). Reads were classified by GOTTCHA using databases comprised of bacteria (species-level: gottcha-speDB-b; strain-level: gottcha-strDB-b) or viruses (species-level: gottcha-speDB-v; strain-level: gottcha-strDB-v), Kraken (kraken_mini), metaphlan and BWA against RefSeq (BWA-mem). All reads that were classified by BWA are presented as a Krona plots, where percentages are the number of reads that map to each organism divided by the total number of classified reads (**B**,**D**).

Three of four taxonomy tools (GOTTCHA, Kraken and BWA) agreed and identified both reads and contigs from the Pseudomonas phage vB_PaeP_130_113 sample as a Pseudomonas phage (Figure 2A). BWA classification results are presented in detail. We present BWA classification results because this tool performs both read and contig-based classification. Only 46.47% of all reads from Pseudomonas phage vB_PaeP_130_113 were classified using taxonomy-based analysis. However, of the reads that were classified, 99% of classified reads were Viruses, all of which were further classified as Pseudomonas phages (Figure 2B). Of the organisms detected by two or more read-based tools, all have >87% identity to the final phage genome. For contig-based community profiling the top hit was Pseudomonas phage vB_Pae-TbilisiM32 (GI:KX711710), which has 95% query coverage and 94% identity to the final phage contig. The total assembly size for Pseudomonas phage vB_PaeP_130_113 was 44,325 bp. The number of reads mapped to phage contig was 203,003 while the number of reads mapped to the whole assembly was 203,005 and the total trimmed reads is 206,222. This amounts to 0.001% potential host sequence. Taken together, these data indicate that the Pseudomonas phage vB_PaeP_130_113 sample passes this checkpoint.

Likewise, three of four taxonomy tools (GOTTCHA, Kraken and BWA) agreed and identified both reads and contigs from the Staphylococcus phage vB_SauM_0414_108 sample as a Staphylococcus phage (Figure 2C). 79.12% of all reads from Staphylococcus phage vB_SauM_0414_108 were classified by BWA. Of the classified reads, 99.99% were classified as Viruses and all viral reads were classified as viruses of *Staphylococcus* at the species level (Figure 2D). The top five organisms classified by read-based taxonomy have >93% identity to the final phage genome. Only two contigs were >700 bp and only the phage contig had over 5× coverage. The top hit for the largest contig, the unfinished phage contig, was Staphylococcus phage JD007 (GI:JX878671), which has 95% query coverage and 97% identity to the final phage genome. The next largest contig (751 bp with under 1.5× coverage) presents no significant similarity to any known sequences in the nr database (megablast). The total assembly size for the Staphylococcus phage vB_SauM_0414_108 sample was 145,555 bp, with the number of reads mapped to phage contig being 338,323 and the number of reads mapped to assembly being 338,360. The total trimmed reads amount to 341,976. These results indicate 0.011% potential host sequence. This phage also passed criteria for contamination-free sequences and does not, by this analysis, appear to exhibit generalized transduction.

### 3.6. Genome Polishing

After genomes passed all the previous twelve checkpoints, the final phage genomes underwent automated gene-calling followed by manual curation of annotations and the final checkpoint:

13. Were any unsettling genes identified by manual annotation?

**Figure 3:**
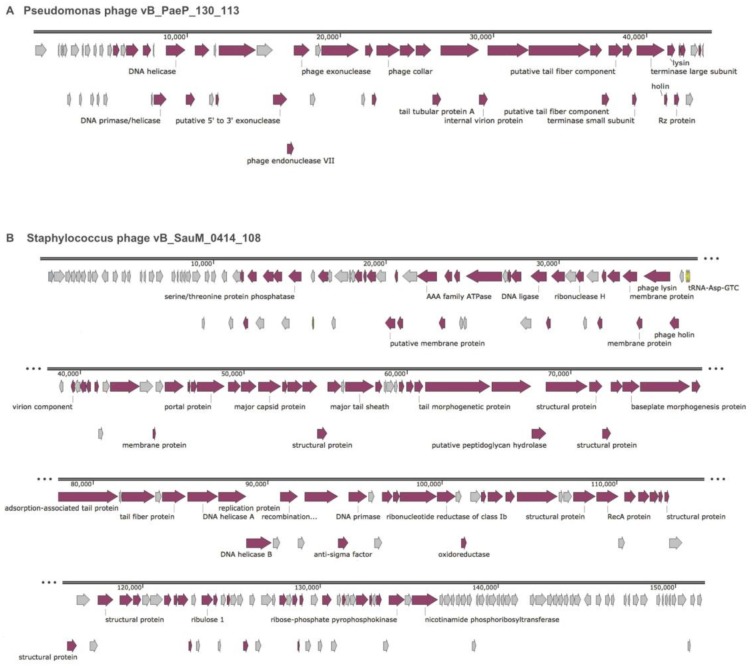
Whole genome maps for finished annotated phage genomes. Annotations for selected predicted open reading frames (ORFs) are presented for Pseudomonas phage vB_PaeP_130_113 (**A**); and Staphylococcus phage vB_SauM_0414_108 (**B**). Mauve colored arrows indicate the ORF has been annotated; grey colored arrows indicate ORFs annotated “hypothetical”; yellow arrows indicate tRNA.

Two major considerations at this step include methods to identify genes and the reliability of annotations. We performed gene calling using RAST and two gene prediction algorithms and compared start and stop sites for each ORF. For each putative ORF, the amino acid sequence was searched against a minimum of four databases (see Methods). Consensus annotations were assigned followed by a final check for any notable genes. The finished genome for Pseudomonas phage vB_PaeP_130_113 contains 57 coding sequences (CDS), 35 with assigned functional annotations, 22 hypothetical, and no tRNA (Table 5, Figure 3A). The final genome for Staphylococcus phage vB_SauM_0414_108 contains 241 CDS, 154 with assigned functional annotations, 87 hypothetical, and four tRNA sequences (Table 5, Figure 3B). No notable or problematic genes were found in either genome.

## 4. Discussion

The promise of phage therapeutics for devising personalized treatment toward AMR microbes demands a reliable and scalable pipeline to characterize phages. Herein we present an analysis workflow along with recommendations for essential checkpoints to assess the genomic safety of phage candidates. This analysis mirrors efforts carried out by our team for phage genomes submitted to the FDA for IND approval. The two phage genomes delivered here are examples of candidates that would pass all safety criteria. We have made our source code publically available at GitHub (https://github.com/BDRD-Genomics). Below we expand on potential issues that arise at important checkpoints and emphasize the need for manual oversight for genomes being considered for human use.

Phage genomes are hyper-mobile and exhibit high mutation rates, thus a finished genome represents a consensus sequence for the distribution of non-identical related progeny. Detecting minor variants is dependent on sequencing technology and population diversity (i.e., quasi-species). Increasing genomic depth of coverage typically provides additional confidence in overcoming sequencing errors and identifying true single nucleotide polymorphisms (SNPs). Previous studies have determined that 400× coverage is recommended to detect minor variants (present at 1% frequency with 99.999% confidence) in order to accurately describe genetic diversity within a viral population [8]. Although dependent on experimental conditions, there exists an upper range limit in which increasing coverage either produces no additional benefit or has deleterious effects. As noted previously, most genomic assemblers use heuristics in order to solve an NP-hard problem (a class of problems which may take exponential time to solve or are unsolvable) in a reasonable period of time. Generally, these heuristics are geared toward lower coverage levels (<100×). Very high coverage may actually increase assembly fragmentation; with low coverage a given error is typically unique but with high enough coverage the same error may be encountered multiple times and assembled. This creates false branches with a de Bruijn graph-based assembler that may be cut, increasing fragmentation. In order to overcome any potential biases introduced by specific assembler heuristics, we employ multiple assemblers (CLC and SPAdes) at multiple depths of coverage, followed by a consensus based approach to determine the most accurate contig. There are a number of freely available open source assemblers (e.g., Velvet, SOAPdenovo, ABySS), which may be substituted for the proprietary product produced by CLC.

Determining a phage’s genomic termini and packaging strategy is necessary for producing the correct nucleotide sequence, selecting the genome start-site, correcting assembly artifacts caused by direct terminal repeats (DTRs), and elucidating the phage’s biology. For example, DNA packaging strategy has implications in a large part of a phage’s life cycle including: initiation, replication, termination and transcriptional regulation [51]. PhageTerm was selected as the tool of choice for analyzing phage termini because of its automated and user-friendly nature [25]. Like other methods that use high-throughput sequence data to ascertain genomic termini, PhageTerm exploits the random nature of DNA fragmentation during library prep and identifies over-represented fragment ends. Genome orientation cannot be inferred from NGS data for *cos* and DTR phages. PhageTerm will leave the orientation the same as the input reference for these types of phages. In this situation, orientation should be determined based on the orientation of terminase gene(s). Similarly, when a phage’s termini cannot be determined, or in the case a phage has no consistent biological termini (circularly permuted phages), it is recommended that the start site be selected relative to the position and orientation of the terminase gene(s). Merril et al. suggest starting circularly permuted phages at, or just upstream from, the large terminase subunit and adjusting the orientation of the genome so it is in the forward direction [26]. However, this often results in the small terminase gene being placed at the opposite end of the genome because it is common for the small terminase subunit to be directly upstream from the gene for the large terminase subunit. Circularly permuted phages put through our workflow start at the small terminase gene so that genes for terminase subunits stay adjacent to each other. The orientation of the genome was adjusted such that it matches the orientation of the small terminase genes. If no small terminase gene can be found, the genome was instead adjusted to the large terminase gene. It is important to note that users should manually inspect the sequence to make sure that the start site does not result in a broken CDS.

Phages that have a high potential to integrate into the chromosomes of their bacterial host are considered undesirable for phage therapy. Our pipeline combines homology-based searches (RAST, PHASTER) and a predictive computational model (PHACTS) to identify risky candidates during the initial viability checkpoint. For annotation-based analysis, integrase, the enzyme that mediates incorporation of phage DNA into bacterial DNA, is the molecular marker used to exclude prophages and phages with temperate potential. Importantly, integrase genes are highly diverse genomic elements, thus relying on sequence similarity to published genomes alone is inadequate for predicting phage lifestyle. PHASTER annotation, for example, only detected 75 of 147 (51%) integrase genes in prophages from *Salmonella enterica* [52]. Moreover, a mutation in a single amino acid residue can render an integrase inactive [53], thus the functional capacity of an integrase is unknown unless it is 100% identical to an experimentally validated annotation. To circumvent the limitations of functional annotation we combine our analysis with PHACTS, a classification algorithm that predicts phage lifestyle based on the entire phage proteome. PHACTS utilizes a novel similarity algorithm and a trained Random Forest classifier to classify phages. These classifications are based on a curated database of phages with annotated lifestyles, which have their proteins aligned against the proteins of the query phage. PHACTS does not always provide clear classifications despite being trained using phages with experimentally proven temperate or lytic lifestyles. It is possible that some discrepancies can be explained by specialized host-phage interactions that govern lifestyle through interference of integrase or other repressor genes (reviewed extensively in [54]). Analysis of repressor genes would be an additional strategy that could add information to phage lifestyle predictions. Another explanation for potentially unclear results from PHACTS could be due to the collection of genomes in the database on which PHACTS is trained. If the query phage has proteins that are similar to those in the database, PHACTS has a higher chance of providing a clear answer. However, if the query phage is fairly unique, it becomes more difficult for the algorithm to make a confident call. The PHACTS training data contains phages that infect *Pseudomonas* and *Staphylococcus* hosts at equal proportions (~50 genomes each), which bolsters confidence in the results presented above. It is important to note that the PHACTS database is limited (zero phages for *Acinetobacter* hosts, for example) and users should be aware that PHACTS predictions are dependent on this database. If users are studying genomes not represented in the database, it is recommended that PHACTS be retrained with a wider variety of phages from a number of different hosts. An additional caveat is that episomal and plasmidial prophages [55,56] might not be detected as such by our analyses unless they exhibit significant nucleotide identity to previously sequenced episomal or plasmidial prophages.

Monitoring the apparent host sequences as a way of assessing potential sample contamination is important for obtaining high quality finished genomes with confidence and ensuring the reproducibility of bioinformatics analysis using raw sequences that are delivered to the FDA as a part of IND-filing. It is also important as a way of monitoring for potential transducing ability. We recommend performing taxonomy classification on all reads and contigs, and, due to the potential uncertainties introduced into the resulting assembly and the qualitative nature of genomic assessments of “safety,” disregarding samples with contamination, even if the host sequence is thought to be introduced downstream of phage purification, such as from sequencing run carry-over or bleed-through among samples multiplexed within a sequencing run. EDGE Bioinformatics provides a well-documented web-based interface to perform classification using four different tools that vary in sensitivity and specificity (see Appendix A; [33,38]). This analysis allows users to generate a confident assessment of the proportion of host sequence and/or of potential contaminants from other sources. The tools are dependent on the curation of the respective databases and underlying algorithms. This means that the number of reads mapping to a particular reference will vary among tools, preventing definitive cut-off values for percent contamination. We recommend running taxonomic classification using multiple tools and focusing on calls where agreement is observed across tools. Importantly, the taxonomy tools in EDGE are designed for classifying prokaryotes and viruses; fungal contaminants will not be classified using this method. At a minimum, we recommended samples undergo read and contig-based analysis using BWA-mem mapping to RefSeq. We also suggest calculating the percentage of reads that map to non-phage contigs. This calculation gives an estimation of how pure a phage prep is and will enable a user to discriminate contamination even if the contaminant is not classified by taxonomy tools (i.e., fungal contamination). For instance, assume that a phage sample had 1,263,276 reads mapped to phage contig and the total number of reads mapped to whole assembly was 1,459,830 out of a total of 1,464,496 quality-controlled reads. In this case, the number of reads mapped to non-phage was 196,554 reads or 13.42%. Another factor to consider is the total genome assembly size, in this example, 6,103,974 bp. Since two facts violate the checkpoints (undesirable percentage of reads not mapping to phage contig and total assembly size much larger than any known phage genome), this phage sample would be abandoned and not analyzed further. When considering total genome assembly size, we may deduce that if the total genome assembly size is much too big to be a phage genome alone, it is likely that it includes several contigs resulting from host bacterial sequences. Based on our experience, if the genome assembly size is larger than 1 Mbp or close to the size of host bacterial genomes (i.e., 3 to 6 Mbp) and if the percentage of non-phage reads is greater than 5%, the quality or purity of the phage prep should be questioned, and/or the possibility of transduction considered.

Prior to intensive annotation, our pipeline employs an initial scan against known deleterious genes, using two methods to screen against VFDB. One method queries the phage contig against VFDB using blastn. We have found that some regions in phage genomes share sequence similarity to hits in VFDB, however these hits present low query coverage and can be rapidly excluded as false positives. For this reason, we have selected to use ShortBRED in combination with VFDB as a second method for screening. The ShortBRED algorithm is used to identify unique and distinguishable protein sequences in VFDB and provides highly specific results. If any positive results are identified using ShortBRED, the phage does not pass as a viable candidate. Users should be cautious of VFDB hits when using BLAST; specifically users should investigate whether identity spans the full protein sequence or if functional domains are represented in the sequence.

Even with that robust screening method in place to assist in detection of deleterious genes, thorough annotation as outlined in our pipeline serves the primary role of assessing phage candidates for safety. Identifying and annotating phage genes remains technically difficult due to small size and lack of known homologues. Additionally, in silico gene-prediction and annotation platforms cater to the molecular underpinnings of bacterial genomes. This means that manual refinement is necessary to checking genes and annotations. Relaxing similarity thresholds of search algorithms may be necessary to fully exclude a gene product as hazardous with confidence. With that being said, it is important to note that assigning a gene product “hypothetical protein” is preferred over a more specific annotation with little evidence when depositing final genomes into public repositories. We recommend reviewing guidelines written by Aziz et al. [9], a thorough overview of how to avoid precarious functional assignments. However, we do want to articulate a major warning for identifying and dismissing “red-flag” annotations deposited without experiential evidence. To exemplify this, note the following two genes: toxin TX1 (Pseudomonas phage TH30) (NCBI reference sequence: YP_009226100) and Acriflavin resistance protein (Pseudomonas phage vB_PaeM_C2-10_Ab02) (NCBI reference sequence: CEF89094). Neither of the aforementioned genes have experimental evidence, nor do any of the homologues, all of which are annotated hypothetical. Moreover, the phage containing the gene annotated “toxin TX1” was administered to animals as a therapeutic phage in preclinical experiments, with no adverse effects. These predicted gene products have no similarity to any other toxins. Finally, in some unique cases protein structure modeling can provide clarity. Open-source computational modeling software, like RaptorX [57] and I-TASSER [30], can be used to predict the integrity of enzymes or identify functions based on similarity to known protein structures rather than sequence similarity in order to refine the granularity of annotations. Similarly, publicly available websites like Superfamily [58] provide a platform for structure based searches using hidden Markov models, which can be useful for predicting the function of distantly related proteins. When submitting phage genomes to the FDA for clinical approval, we recommend providing annotation results from all databases and the date databases were accessed. By using orthogonal approaches and combining the results through manual curation we can say with confidence that no known deleterious genes are encoded within candidate phage genomes that pass through our checkpoints, but we cannot exclude the possibility that unknown, previously unsequenced or uncharacterized, deleterious genes could exist that we might not detect through in silico safety analyses.

Another caveat is that in this study we have not addressed the utility of different sequencing chemistries and platforms. In the course of this work we have mainly utilized Illumina short read sequencing technology, although for some genomes we have attempted to resolve potential assembly artifacts through the use of long reads. Short read technologies typically (1) have inherently low error rates and (2) due to the high throughput, resulting deep coverage [59]. On the contrary, long read sequencing platforms such as the Oxford MinION and PacBio hold promise for resolving terminal repeats and other ambiguities in phage genome assemblies, but the combination of higher error rates and less deep coverage [60,61,62] is likely to make error correction with short reads a necessity in many cases for at least the near future. The pipeline presented in this manuscript would require adjustment to be suitable for long reads, particularly the QC and assembly steps, and subsampling would likely not be required for long reads. However, the downstream processes and checkpoints regarding lifestyle, annotation, and potentially dangerous genes, would remain the same.

In conclusion, we present to the phage therapeutic community a set of guidelines to enable not only the production of high quality phage genomes and but also predictions of phage suitability for therapeutic use. Many of the steps involved are intuitive but some are not. Most of these steps would be employed in genome production and characterization standards for a variety of organisms but in most cases outside of phage therapy, not all of these steps would be required. In many cases there are examples of published genomes that would have benefited from some of these steps. There is currently no single pipeline that is completely automated from end-to-end and would accomplish all the suitability checks and verifications inherent to this type of work but we have made our source code available to the general public and have documented herein the types of human intervention involved and the logic that is applied. We have also made the raw sequence data as well as the polished final products available in public databases for use by others. This work leverages previously developed standards (that were primarily focused on producing genomic data for pathogens or for vaccine candidates [14]) to create the first fully described and published standard for genomes of therapeutic viruses. The approach presented herein should enable researchers to characterize potential therapeutic phages fully prior to IND submission.

## 5. Conclusions

In conclusion, the pipeline and checkpoints presented here represent a necessary first step toward widespread use of phage therapy in the US. The aim is to produce fully assembled, error-free, well-annotated genomes for lytic phages that do not encode genes likely to promote toxicity or AMR and to do so through a combination of best available tools, well-defined thresholds and necessary human interventions. In support of this movement, we provide the pipeline and our internal thresholds to the scientific community, along with datasets that can be used for training purposes.

## Figures and Tables

**Table 1:** Databases curated with virulence factor and antimicrobial resistance genes.

Database	# Of Genes in Database	Last Updated ^1^	Database Source
ShortBRED VF ^2^	26,187	July 2017	https://huttenhower.sph.harvard.edu/shortbred
ShortBRED AR ^3^	932	July 2017	https://huttenhower.sph.harvard.edu/shortbred
Virulence Factor DataBase (VFDB)	30,246	February 2018	http://www.mgc.ac.cn/VFs/main.htm
Comprehensive Antibiotic Resistance Database (CARD)	2514	February 2018	https://card.mcmaster.ca/download

^1^ Last update available for public download. Database download dates for analyses in this manuscript are described in Materials and Methods. ^2^ Database built using Victors, VFDB and MvirDB. ^3^ Database built using CARD.

**Table 2:** Sequencing and Assembly Statistics.

Pipeline Output	Pseudomonas Phage vB_PaeP_130_113	Staphylococcus Phage vB_SauM_0414_108
Total Reads	206,222	347,594
Reads Pass FaQCs (%)	98.82	98.38
Reads Pass CLC (%)	96.97	94.03
Reads sub-sampled (#)	50,000	50,000
SPAdes all reads ^1^	1	2
CLC all reads ^1^	1	1
SPAdes subsampled ^1^	1	1
CLC subsampled ^1^	1	1
SPAdes all reads ^2^	43,742	141,507
CLC all reads ^2^	43,742	141,334
SPAdes subsampled ^2^	43,742	141,331
CLC subsampled ^2^	43,742	141,330

^1^ Number of contigs >700 base pairs long. ^2^ Length of largest contig (bp), SPAdes assembly artifacts removed.

**Table 3:** Genomic termini statistics.

Phage	Class ^1^	DTR Region Length	Start, End τ Metric ^2^	Coverage in DTR Region	Coverage Outside of DTR Region
Pseudomonas phage vB_PaeP_130_113	Short DTR	463 bp	0.63, 0.64	1018.0×	634.9×
Staphylococcus phage vB_SauM_0414_108	Long DTR	10,296 bp	0.75, 0.55	753.1×	343.9×

Above metrics are determined by PhageTerm. ^1^ One of the following: 5′ *cos*, 3′ *cos*, Short DTR, Long DTR, headful (with or without *pac* site detected), Mu-like, or unknown. ^2^ τ in forward direction for first nucleotide of DTR region, τ in reverse direction for last nucleotide of DTR region.

**Table 4:** Phage lifestyle assessment.

Phage	PHACTS Lytic Score	PHACTS Temperate Score	PHACTS Standard Deviation	PHASTER Integrase	RAST Integrase	NCBI Annotated Integrase
Pseudomonas phage vB_PaeP_130_113	0.66	0.34	0.073	No	No	N/A
Pseudomonas phage DL62 (GI:KR054031)	0.73	0.26	0.117	No	No	No
Pseudomonas phage vB_PaeS_PMG1 (GI:NC_016765)	0.42	0.58	0.042	Yes	Yes	Yes
Staphylococcus phage vB_SauM_0414_108	0.60	0.40	0.082	No	No	N/A
Staphylococcus phage K (GI:KF76114)	0.59	0.41	0.107	No	No	No
Staphylococcus phage phiSaus-IPLA88 (GI:NC_011614)	0.28	0.72	0.048	Yes	Yes	Yes

N/A = Not applicable due to in house anntoation.

**Table 5:** Finished genome details.

Phage	Size (bp)	%GC	CDS (#)	Genes with Functional Annotation (#)	Hypothetical Genes (#)	tRNA (#)	Assigned Family
Pseudomonas phage vB_PaeP_130_113	44,205	62.4	57	35	22	0	Podoviridae
Staphylococcus phage vB_SauM_0414_108	151,627	30.4	241	154	87	4	Myoviridae

## Supplementary Materials

The following are available online at http://www.mdpi.com/1999-4915/10/4/188/s1, Table S1: Parameters employed.

## Appendix A

EDGE (Enabling the Democratization of Genomics Expertise) is a bioinformatics platform with a user-friendly interface which provides access to cutting edge bioinformatics tools. Of the seven modules available in EDGE, we use four modules to analyze phage sequences. The pre-processing module performs quality control and read trimming; we changed trim quality level to 30, average quality cutoff to 30 and “N” base cutoff to 2. The assembly and annotation module assembles the reads into contigs, performs gene calling using Prodigal and annotation with Prokka; we changed the assembler to SPAdes and Prokka kingdom to Viruses. The taxonomy classification module uses multiple tools to assign taxonomic classifications to reads and contigs; we use default parameters. The gene family module, added in EDGE v1.5, searches specially curated databases for virulence factors and antibiotic resistance genes in our reads and predicted ORFs; we use default parameters.

EDGE code (including the Gene Family analysis) is available at:

- https://github.com/LANL-Bioinformatics/EDGE.

EDGE documentation is available at:

- https://edge.readthedocs.io.

EDGE tutorial is available at:

- https://www.youtube.com/playlist?list=PL7DNo6h5wJsTh2l2GK3N86Imb-9fYQFfH
