# Supplementary material for: Characterizing Phage Genomes for Therapeutic Applications
Source: Viruses. 2018 Apr 10;10(4):188. doi: 10.3390/v10040188 (PMC5923482; doi:10.3390/v10040188)
Supplement: Supplementary file 1 [file viruses-10-00188-s001.pdf]

EDGE Trimming	- Trim Quality Level: 30<br>- Average Quality Cutoff: 30<br>- Minimum Read Length: 50<br>- “N” Base Cutoff: 2<br>- Low complexity Filter Ratio: 0.85<br>- Adapter FASTA: None<br>- Cut #bp from 5’ end: 0<br>- Cut #bp from 3’ end: 0
CLC Trimming	- Ambiguous limit: 2<br>- Quality limit: 0.001<br>- Remove 5’ terminal nucleotides: 0<br>- Remove 3’ terminal nucleotides: 0<br>- Maximum number of nucleotides in reads: 1000<br>- Minimum number of nucleotides in reads: 50<br>- Discard short reads
EDGE Assembly	- Assembler: SPAdes<br>- Minimum Kmer Length: 31<br>- Maximum Kmer Length: 121<br>- Step size: 20<br>- Minimum Contig Length: 200<br>- Validation Aligner: Bowtie 2
CLC Assembly	- Mapping mode: Map reads back to contigs (slow)<br>- Update Contigs<br>- Mismatch cost: 2<br>- Insertion cost: 3<br>- Deletion cost: 3<br>- Colorspace error cost: 3<br>- Length fraction: 0.5<br>- Similarity fraction: 0.8<br>- Colorspace alignment<br>- Alignment mode: global<br>- Match mode: random<br>- Bubble size: Automatic<br>- Word size: 64<br>- Minimum contig length: 200<br>- Perform scaffolding<br>- Auto-detect paired distances
PhageTerm	Paired reads<br>-s: 20<br>-d: 20<br>-m: 250
Contamination Check	- Must be less than 5% of reads mapping to host
VF/AR/Integrase Presence	

PHACTS	--classes: classes_lifestyle<br>--replicates: 20<br>--exclude: None<br>--num_cases: 50<br>--percent: 1<br>--variables: 600<br>--short: 0
High Quality Genome	- 90% of reads must map back to final genome<br>- 100X coverage for complete genomes<br>- ~400X coverage for finished genomes
